# The Needs of the Queen's Hospital for Children

**Published:** 1913-05-31

**Authors:** 


### The Needs of the Queen's Hospital for Children.

Sir Herbert Tree, as a member of the committee, in an interesting review of the work and development of the Queen's Hospital for Children, Hackney Road, upon the occasion of the annual meeting of governors on the 19th inst., at which he presided in the absence of the Earl of Shaftesbury, referred to the fact that the Queen's was one of the few London hospitals that enjoyed the privilege of being served on the management board by ladies. He said that he thought it was especially appropriate that the Queen's Hospital should be so served, seeing that it owed its foundation to two Quaker ladies engaged in missionary and nursing work amongst the poor of Bethnal Green. One of the founders, Miss Mary E. Phillips, is still living. It now possessed 164 beds, as compared with the ten with which it commenced forty-five years ago. The hospital had been a pioneer in many directions, and the fact that it was the first hospital of its kind to make arrangements for the complete dental treatment of children was an outstanding instance of this.

The Treasurer, Mr. Maurice Rüffer, in moving the adoption of the report and accounts, said that, although the hospital had paid its way in 1912, the accumulated deficits on the general account of previous years, which had been brought forward, amounted to £1,260. This deficit had now grown to £2,500, which was due to a falling off in receipts to the extent of £1,200. The committee felt that an appeal was urgently necessary, and were asking for £3,500, but he wished to emphasise the fact that this sum would by no means cover the requirements. The annual expenditure upon the hospital and its Bexhill branch was about £15,500, and the reliable income was only £1,000. Their indebtedness on mortgage loans amounted to £3,500, and, after allowing for certain legacies which were due to be paid, they would be faced with a debt of about £5,500. The Chairman of the House Committee, Mr. Charles Port, who seconded the motion, pointed out that in the course of ten years the accommodation of the hospital had been increased from 57 to 164 beds, including 30 at Bexhill, and that this progress had been achieved without increasing the total debt to more than it was twenty years ago. It is worthy of note that the formal business which followed the adoption of the report included the addition of the new Chief Rabbi, Dr. J. H. Hertz, to the list of distinguished vice-presidents of the hospital. The proceedings concluded with a vote of thanks to Sir Herbert Tree for presiding, which was proposed by Mr. Joseph Meller, and seconded by Mr. P. Lockhart Mummery, F.R.C.S., to which Sir Herbert Tree briefly responded, remarking that the action of the Treasurer, Mr. Rüffer, who had just given a donation of £50, was one which he hoped would be followed by many. Mr. Meller, deputy-chairman of the house committee, said that the pressure on the hospital was so great that they had to utilise not only cots but cradles.

